# Supplementary material for: Intercalation of Mn in graphene/Cu(111) interface: insights to the electronic and magnetic properties from theory
Source: Sci Rep. 2020 Dec 10;10:21684. doi: 10.1038/s41598-020-78583-w (PMC7729943; doi:10.1038/s41598-020-78583-w)
Supplement: Supplementary file 1 — Supplementary Informtaion. [file 41598_2020_78583_MOESM1_ESM.pdf]

Supplementary information for

Intercalation of Mn in graphene/Cu(111) interface: insights to the electronic and magnetic properties from theory

Qilin Guo[1], Yuriy Dedkov[1,*], and Elena Voloshina[1,+]

[1]Department of Physics, Shanghai University, Shangda Road 99, 200444 Shanghai, China
[*]dedkov@shu.edu.cn
[+]voloshina@shu.edu.cn

List of Tables and Figures:

Tab. S1: Results for the relaxed gr/Cu(111). ΔE (in meV/u.c.) is the relative total energies per graphene (1×1) unit cell (u.c.) with respect to the energetically most favourable structure; E_{int} (in meV/u.c.) is the interaction energy, defined as $E_{\text{int}} = E_{\text{gr/metal}} - (E_{\text{gr}} + E_{\text{metal}})$, where $E_{\text{gr/metal}}$ is the total energy of the graphene/metal system, and E_{gr} and E_{metal} are the energies of the fragments at the same coordinates as in the graphene/metal system; d_0 (in Å) is the distance between the graphene overlayer and the interface metal layer; d_1 (in Å) is the mean distance between the interface metal layer and the second metal layer; d_2 (in Å) is the mean distance between the second and third metal layers.

Tab. S2: Results for the relaxed gr/Mn/Cu(111) with different magnetic configuration (FM=ferromagnetic, AFM=anti-ferromagnetic). ΔE (in meV/u.c.) is the relative total energies per graphene (1×1) unit cell (u.c.) with respect to the energetically most favourable structure; E_{int} (in meV/C-atom) is the interaction energy, defined as $E_{\text{int}} = E_{\text{gr/metal}} - (E_{\text{gr}} + E_{\text{metal}})$, where $E_{\text{gr/metal}}$ is the total energy of the graphene/metal system, and E_{gr} and E_{metal} are the energies of the fragments at the same coordinates as in the graphene/metal system; d_0 (in Å) is the mean distance between the graphene overlayer and the interface metal layer; d_1 (in Å) is the mean distance between the interface metal layer and the second metal layer; d_2 (in Å) is the mean distance between the second and third metal layers; m_{C} and m_{Mn} (in μ_{B}) are the interface/surface carbon atoms and manganese atoms spin magnetic moments (cf. Fig. S3a).

Tab. S3: Results for the relaxed gr/Cu_2Mn/Cu(111) (model B) with different magnetic configuration (FM and AFM). Here ΔE (in meV/s.c.) is the relative total energies per graphene (3×3) super cell (s.c.) with respect to the energetically most favourable structure. E_{int} (in meV/C-atom) is the interaction energy, defined as $E_{\text{int}} = E_{\text{gr/metal}} - (E_{\text{gr}} + E_{\text{metal}})$, where $E_{\text{gr/metal}}$ is the total energy of the graphene/metal system, and E_{gr} and E_{metal} are the energies of the fragments at the same coordinates as in the graphene/metal system; d_0 (in Å) is the mean distance between the graphene overlayer and the interface metal layer; d_1 (in Å) is the mean distance between the interface metal layer and the second metal layer; d_2 (in Å) is the mean distance between the second and third metal layers; m_{C} and m_{Mn} (in μ_{B}) are the interface/surface carbon atoms and manganese atoms spin magnetic moments (cf. Fig. S3b).

Fig. S1: Top view of the crystallographic structures of graphene/Cu(111): (a) FH - the C atoms are placed directly above the Cu atoms of the third layer (*fcc* site) and the second layer (*hcp* site); (b) TH - the C atoms are placed directly above the Cu atoms of the first layer (*top* site) and the second layer (*hcp* site); (c) TF - the C atoms are placed directly above the Cu atoms of the first layer (*top* site) and the third layer (*fcc* site). The graphene units cell is marked with the blue rhombus.

Fig. S2: Top view of the crystallographic structures of graphene/Mn/Cu(111) where the Mn atoms can be located at the *fcc* or *hcp* site of Cu(111) surface, respectively while the two inequivalent carbon atoms of graphene can adopt the FH, TH or TF configuration. The graphene units cell is marked with the blue rhombus.

Fig. S3: Lowest energy magnetic structures of (a) graphene/Mn/Cu(111) and (b) graphene/Cu_2Mn/Cu(111) system (model B).

Fig. S4: Surface alloy model of graphene/Cu_2Mn/Cu(111) system (model B) with *hcp* lattice, where the first layer of Cu(111) slab is replaced by Cu_2Mn surface alloy and the two inequivalent carbon atoms of graphene can adopt the FH, TH or TF configuration.

Fig. S5: Spin-resolved band structures obtained after unfolding procedure for the graphene (1×1) primitive cell for (a) graphene/Cu_2Mn/Cu(111) - model A and (b) graphene/Cu_2Mn/Cu(111) - model B in their energetically most favourable structures.

Table S1. Results for the relaxed gr/Cu(111). ΔE (in meV/u.c.) is the relative total energies per graphene (1×1) unit cell (u.c.) with respect to the energetically most favourable structure; E_{int} (in meV/C-atom) is the interaction energy, defined as $E_{\text{int}} = E_{\text{gr/metal}} - (E_{\text{gr}} + E_{\text{metal}})$, where $E_{\text{gr/metal}}$ is the total energy of the graphene/metal system, and E_{gr} and E_{metal} are the energies of the fragments at the same coordinates as in the graphene/metal system; d_0 (in Å) is the distance between the graphene overlayer and the interface metal layer; d_1 (in Å) is the mean distance between the interface metal layer and the second metal layer; d_2 (in Å) is the mean distance between the second and third metal layers.

Structure	ΔE	E_{int}	d_0	d_1	d_2	Reference
TF	0.00	−92	3.03	2.10	2.09	Fig. S1a
TH	2.26	−92	3.03	2.11	2.09	Fig. S1b
FH	11.89	−87	3.07	2.11	2.09	Fig. S1c

Table S2. Results for the relaxed gr/Mn/Cu(111) with different magnetic configuration (FM=ferromagnetic, AFM=anti-ferromagnetic). ΔE (in meV/u.c.) is the relative total energies per graphene (1×1) unit cell (u.c.) with respect to the energetically most favourable structure; E_{int} (in meV/C-atom) is the interaction energy, defined as $E_{\mathrm{int}} = E_{\mathrm{gr/metal}} - (E_{\mathrm{gr}} + E_{\mathrm{metal}})$, where $E_{\mathrm{gr/metal}}$ is the total energy of the graphene/metal system, and E_{gr} and E_{metal} are the energies of the fragments at the same coordinates as in the graphene/metal system; d_0 (in Å) is the mean distance between the graphene overlayer and the interface metal layer; d_1 (in Å) is the mean distance between the interface metal layer and the second metal layer; d_2 (in Å) is the mean distance between the second and third metal layers; m_{C} and m_{Mn} (in μ_{B}) are the interface/surface carbon atoms and manganese atoms spin magnetic moments (cf. Fig. S3a).

Magnetic state	Structures gr	Structures Mn	ΔE	E_{int}	d_0	d_1	d_2	m_{Mn}	m_{C}	Reference
FM	FH	hcp	110	—	1.98	2.14	2.10	1.62	−0.02/−0.01	Fig. S2d
		fcc	98	—	1.98	2.14	2.10	1.63	−0.01/−0.02	Fig. S2a
	TF	hcp	717	—	1.93	2.13	2.10	1.68	−0.02/−0.02	Fig. S2e
		fcc	58	−360	1.98	2.13	2.10	1.62	−0.01/−0.02	Fig. S2b
	TH	hcp	67	—	1.98	2.13	2.11	1.61	−0.01/−0.02	Fig. S2f
		fcc	713	—	1.93	2.12	2.10	1.70	−0.02/−0.02	Fig. S2c
AFM	FH	hcp	217	—	2.01	2.15	2.10	±0.04	0.00/0.00	Fig. S2d
		fcc	194	—	1.97	2.10	2.09	±0.02	0.00/0.00	Fig. S2a
	TF	hcp	302	—	3.22	2.18	2.11	±2.89	0.00/0.00	Fig. S2e
		fcc	0	−237	2.01	2.14	2.10	±2.07	±0.01/±0.03	Fig. S2b
	TH	hcp	212	—	2.01	2.15	2.11	±0.07	0.00/0.00	Fig. S2f
		fcc	294	—	3.29	2.19	2.11	±2.90	0.00/0.00	Fig. S2c

Table S3. Results for the relaxed gr/Cu_2Mn/Cu(111) (model B) with different magnetic configuration (FM and AFM). Here ΔE (in meV/s.c.) is the relative total energies per graphene (3×3) super cell (s.c.) with respect to the energetically most favourable structure. E_{int} (in meV/C-atom) is the interaction energy, defined as $E_{int} = E_{gr/metal} - (E_{gr} + E_{metal})$, where $E_{gr/metal}$ is the total energy of the graphene/metal system, and E_{gr} and E_{metal} are the energies of the fragments at the same coordinates as in the graphene/metal system; d_0 (in Å) is the mean distance between the graphene overlayer and the interface metal layer; d_1 (in Å) is the mean distance between the interface metal layer and the second metal layer; d_2 (in Å) is the mean distance between the second and third metal layers; m_C and m_{Mn} (in μ_B) are the interface/surface carbon atoms and manganese atoms spin magnetic moments (cf. Fig. S3b).

	Magnetic state	ΔE	E_{int}	d_0	d_1	d_2	m_{Mn}	m_C	Reference
FH	AFM	106	—	3.15	2.18	2.10	$\pm3.69/\pm3.72/\pm3.70$	0.00	Fig. S4a
	FM	54	—	3.11	2.17	2.09	3.63	0.00	
TF	AFM	34	—	3.10	2.17	2.10	$\pm3.67/\pm3.69/\pm3.67$	0.00	Fig. S4b
	FM	0	−89.23	3.05	2.16	2.09	3.58	0.00	
TH	AFM	28	−87.28	3.10	2.17	2.10	$\pm3.66/\pm3.69/\pm3.67$	0.00	Fig. S4c
	FM	2	−88.95	3.05	2.16	2.09	3.58	0.00	

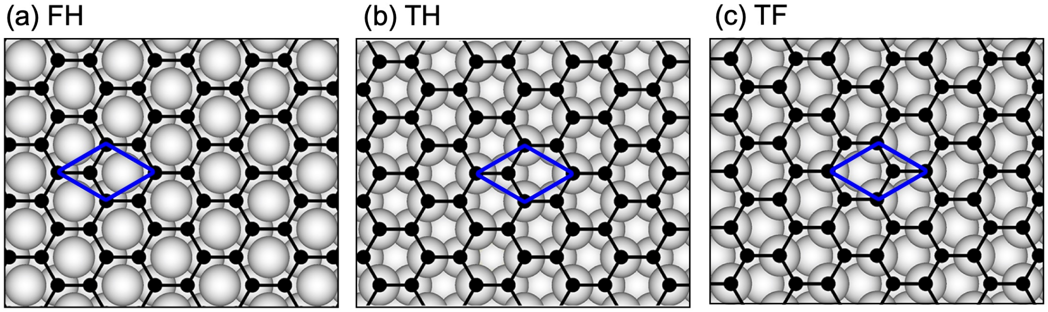

Figure S1. Top view of the crystallographic structures of graphene/Cu(111): (a) FH - the C atoms are placed directly above the Cu atoms of the third layer (*fcc* site) and the second layer (*hcp* site); (b) TH - the C atoms are placed directly above the Cu atoms of the first layer (*top* site) and the second layer (*hcp* site); (c) TF - the C atoms are placed directly above the Cu atoms of the first layer (*top* site) and the third layer (*fcc* site). The graphene units cell is marked with the blue rhombus.

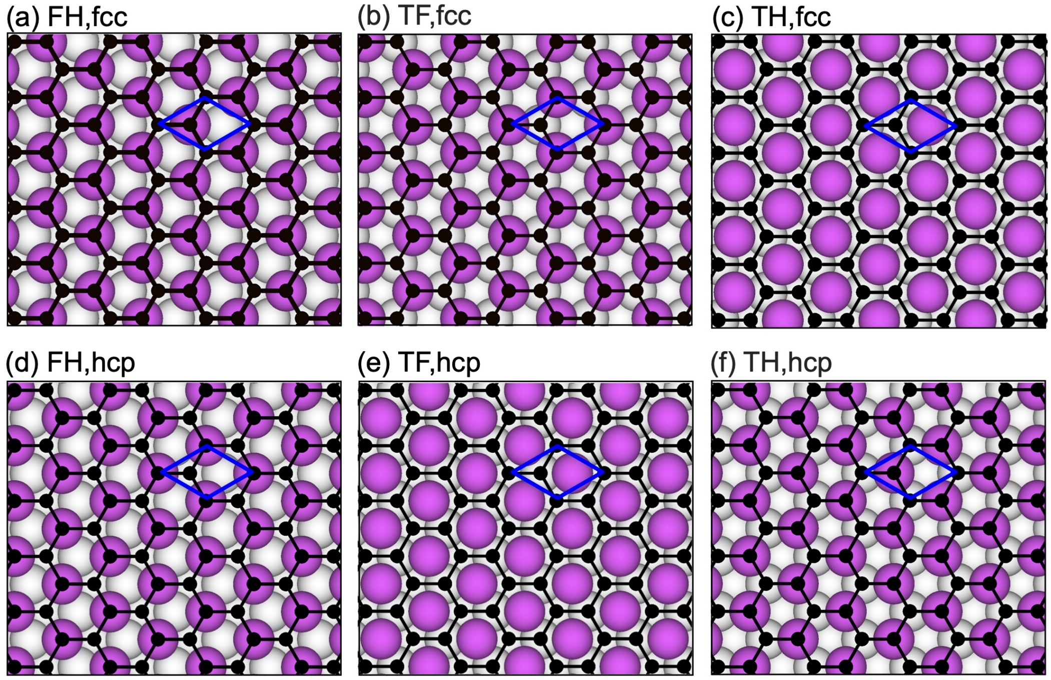

Figure S2. Top view of the crystallographic structures of graphene/Mn/Cu(111) where the Mn atoms can be located at the *fcc* or *hcp* site of Cu(111) surface, respectively while the two inequivalent carbon atoms of graphene can adopt the FH, TH or TF configuration. The graphene units cell is marked with the blue rhombus.

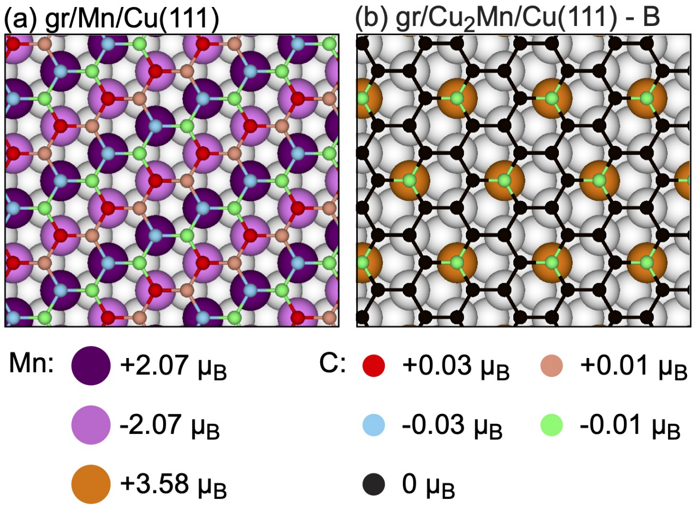

Figure S3. Lowest energy magnetic structures of (a) graphene/Mn/Cu(111) and (b) graphene/Cu_2Mn/Cu(111) system (model B).

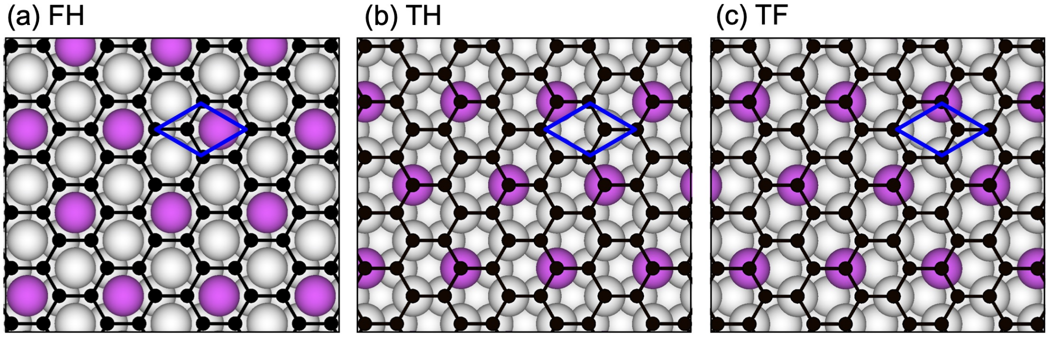

Figure S4. Surface alloy model of graphene/Cu_2Mn/Cu(111) system (model B) with *hcp* lattice, where the first layer of Cu(111) slab is replaced by Cu_2Mn surface alloy and the two inequivalent carbon atoms of graphene can adopt the FH, TH or TF configuration.

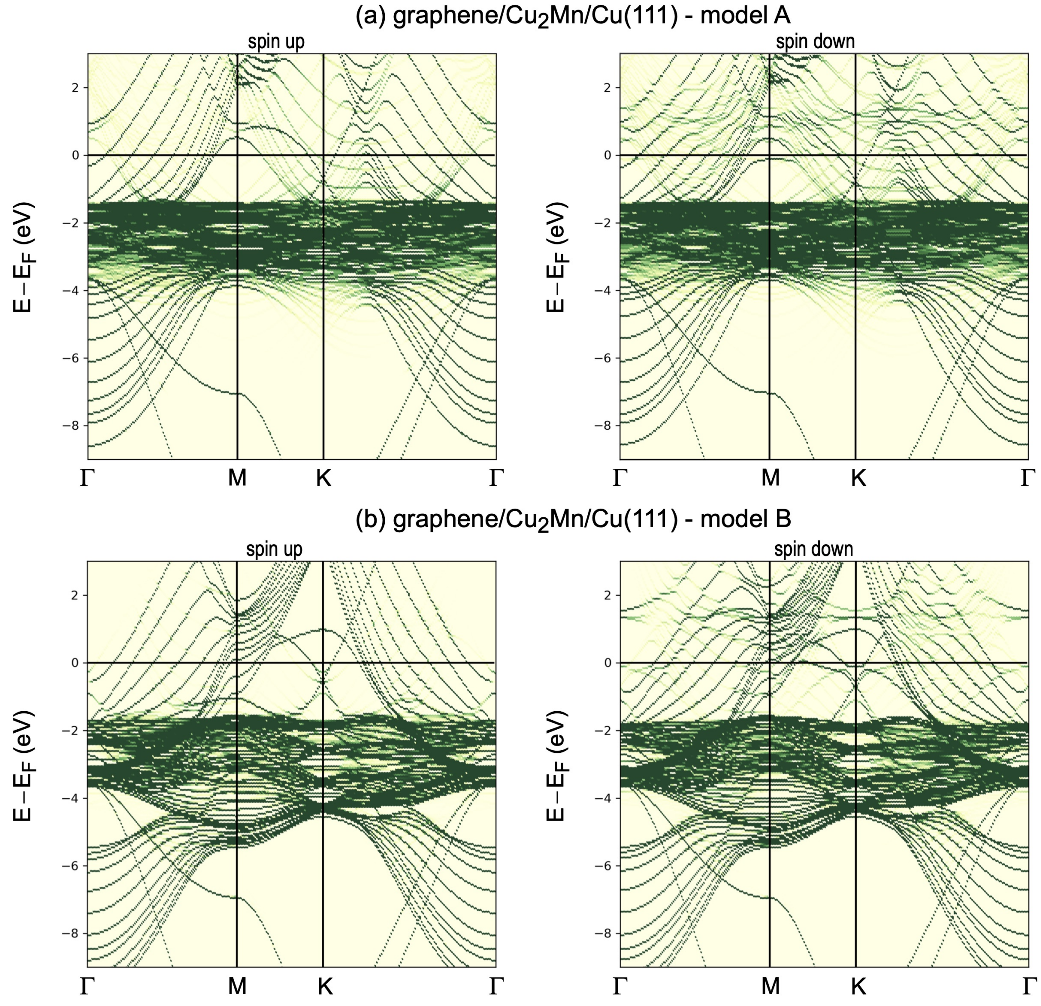

Figure S5. Spin-resolved band structures obtained after unfolding procedure for the graphene (1×1) primitive cell for (a) graphene/Cu_2Mn/Cu(111) - model A and (b) graphene/Cu_2Mn/Cu(111) - model B in their energetically most favourable structures.
